# Compassionate self-talk enhances autonomic flexibility during cognitive stress in generalized anxiety disorder: a randomized controlled trial with HRV evidence

**DOI:** 10.3389/fpsyt.2026.1795990

**Published:** 2026-06-16

**Authors:** Lijun Sun, Yonghui Shen, Ying Wang, Xianwei Che, Yi Lei, Xi Luo

**Affiliations:** 1 Affiliated Mental Health Center and Hangzhou Seventh People’s Hospital, Zhejiang University School of Medicine, Hangzhou, China; 2 Centre for Cognition and Brain Disorders, The Affiliated Hospital of Hangzhou Normal University, Hangzhou, China; 3 Institute for Brain and Psychological Sciences, Sichuan Normal University, Chengdu, China; 4 School of Nursing, Hangzhou Medical College, Hangzhou, China

**Keywords:** autonomic nervous system, cognitive stress, generalized anxiety disorder, heart rate variability, self-compassion

## Abstract

Generalized anxiety disorder (GAD), characterized by persistent and excessive worry, exhibits high treatment resistance. While self-compassion training shows promise in alleviating anxiety, its acute effects on stress-related autonomic regulation in GAD remain unclear. This randomized controlled trial examined whether a single-session compassionate self-talk protocol improves emotional and autonomic stress responses in GAD, focusing on high-frequency heart rate variability (HF-HRV) as an index of parasympathetic engagement. Forty-six clinically diagnosed GAD participants were randomly assigned to either a compassionate self-talk group (*n* = 23) or a neutral self-talk control (*n* = 23). Participants completed a cognitive stress task (time-limited Raven’s matrices with evaluative feedback) with continuous electrocardiogram (ECG) recording. State anxiety (STAI-S), perceived stress, state self-compassion (SSCS), and affect (PANAS) were assessed pre- and post-intervention. The self-compassion group showed greater reductions in perceived stress (*t _(44)_* = -2.09, *p* = 0.043, *d* = 0.62) and increased positive affect (*t _(44)_* = 2.29, *p* = 0.028, *d* = 0.67), though no significant group differences emerged for state anxiety or state self-compassion. During stress exposure, HF-HRV was significantly higher in the self-compassion group (*t _(37)_* = 2.15, *p _corrected_* = 0.038) during a late post-feedback interval. Correlational analyses revealed that increased state self-compassion correlated with reduced stress (*r* = -0.54, *p* = 0.001) and anxiety (*r* = -0.45, *p* = 0.010). These findings suggest that brief compassionate self-talk may improve subjective stress coping in GAD and may be associated with altered event-related autonomic dynamics during cognitive stress. Given the modest sample and ultra-short HRV segments, physiological interpretations warrant a cautious approach. Future studies may test whether combining self-compassion training with HRV biofeedback optimizes intervention efficacy in clinical settings.

**Clinical trial registration:**

## 1 Introduction

Self-compassion, defined as treating oneself with kindness and understanding during stressful experiences (1), enhances adaptive coping strategies and promotes mental health resilience (2–5). Emerging evidence further links self-compassion to physiological flexibility, particularly through higher heart rate variability (HRV) — a biomarker reflecting parasympathetic nervous system activity that supports stress regulation (6, 7). For instance, individuals with elevated self-compassion exhibit greater HRV during acute stressors (8, 9), suggesting a psychophysiological pathway for its therapeutic effects (10).

Generalized anxiety disorder (GAD), characterized by persistent worry and autonomic dysregulation (11), presents a critical test case for investigating self-compassion interventions. While multi-week programs (e.g., 8-session Mindful Self-Compassion) —which typically blend multiple therapeutic components such as mindfulness, loving-kindness, and psychoeducation— demonstrate efficacy in reducing GAD symptoms (12), their prolonged duration limits accessibility for patients with financial or time constraints (13–15). This gap is particularly salient given evidence that self-compassion mediates anxiety reduction in GAD treatments (16). Recent advances in brief (< thirty minutes) self-compassion inductions offer a pragmatic solution. Single-session writing protocols effectively elevate state self-compassion (17) and buffer stress reactivity in socially anxious populations (18), yet no studies have tested acute effects of single-session self-compassion exercises on both emotional and autonomic stress responses in GAD. The physiological mechanisms (e.g., HRV dynamics) underlying these effects remain uncharacterized.

This randomized controlled trial addressed these gaps by investigating a 15-minute writing-based compassionate self-talk protocol (19, 20) in adults with DSM-5 diagnosed GAD. We measured pre-post changes in state anxiety (STAI-S), perceived stress (VAS), affect (PANAS), and state self-compassion (SSCS), followed by HRV assessment during a cognitive stressor (time-limited Raven’s matrices under evaluative threat). We hypothesized that, relative to active control, the self-compassion group would exhibit: (a) reduced negative affect, state anxiety, and perceived stress; (b) increased positive affect and state self-compassion; (c) enhanced HF-HRV during stress exposure – suggesting parasympathetic-mediated resilience.

## 2 Materials and methods

### 2.1 Participants

A power analysis was performed using G*Power 3.1 (21) (*t* tests, Mean: difference between two independent means, two groups, *α* = 0.05, power of 0.8) based on the medium effect sizes in a similar study (18). The results showed that a total sample of no fewer than 42 participants was required. We recruited 46 participants to account for potential attrition. Adults meeting DSM-5 criteria for generalized anxiety disorder (GAD) via the Mini-International Neuropsychiatric Interview (M.I.N.I (22); were recruited through community advertisements. Inclusion criteria required: (a) age 18–65 years; (b) Hamilton Anxiety Rating Scale (HAM-A) score ≥14; (c) Chinese literacy. Exclusion criteria were: (a) alcohol or substance abuse; (b) schizophrenia or bipolar disorder; (c) severe physical illness, cognitive impairment or hearing impairment; (d) risk of self-harm or suicide. Participants were randomized to a self-compassion (SC: *n* = 23, 6 males, age 38.28 ± 7.69) or control group (*n* = 23, 6 males, age 29.7 ± 9.47). For electrocardiograms (ECG) analyses, the final analyzable sample (*n* = 18 SC, *n* = 21 control) resulted from technical exclusions due to motion artifacts during stress tasks (*n* = 4) and participant withdrawal (*n* = 3). The present study was conducted as a nested experimental sub-study within a larger randomized controlled trial (23) (ChiCTR.org.cn, Registration No. ChiCTR2100053374) comparing self-compassion and mindfulness interventions for GAD. The Ethics Committee of Hangzhou Seventh People’s Hospital approved all procedures (No. 2021067).

### 2.2 Experimental design and procedure

**Figure 1:**
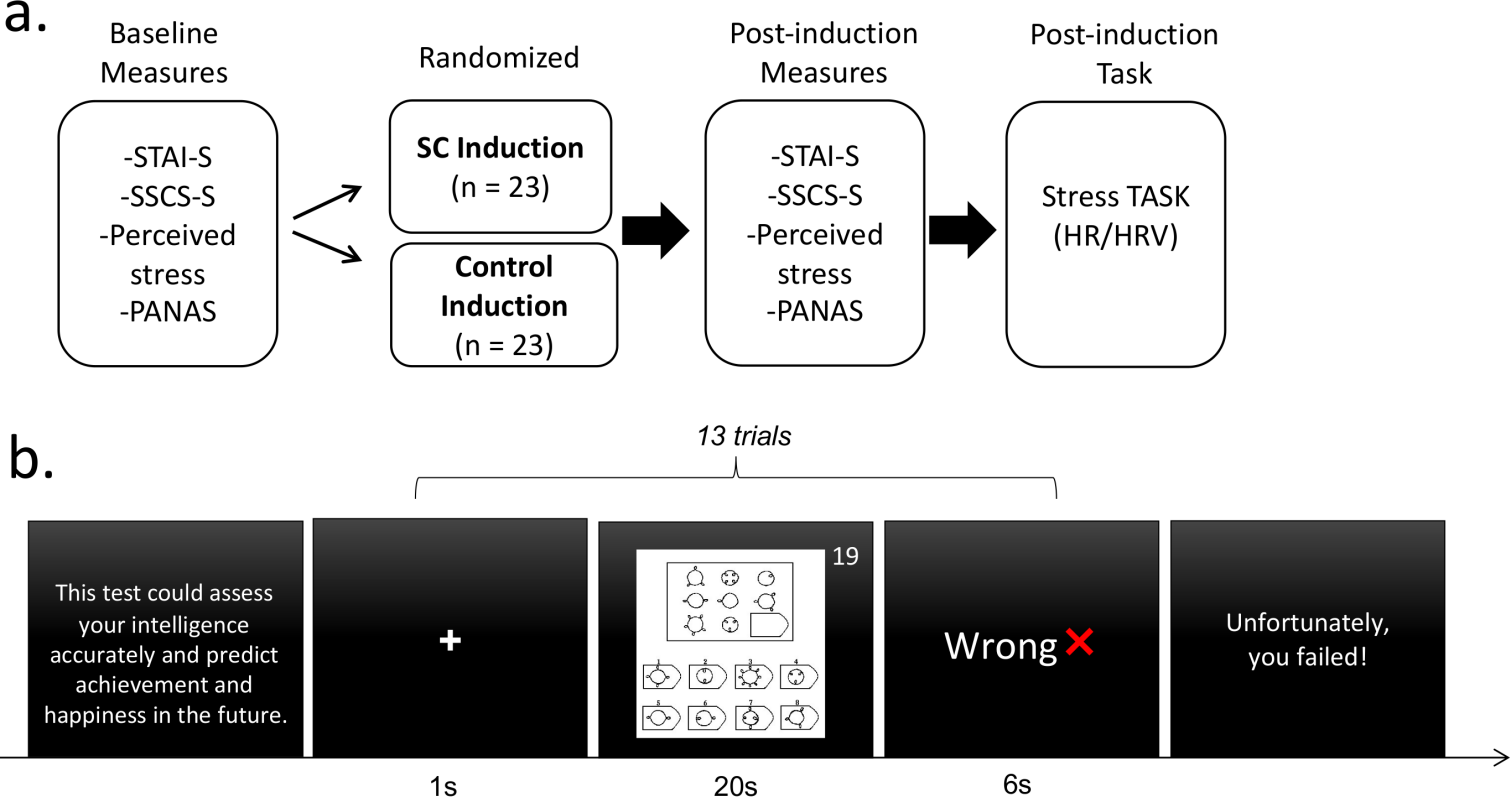
Study design and procedure. **(a)** Schematic of study phases: Baseline assessment, intervention, post-assessment, and stress task with ECG recording. **(b)** Structure of the cognitive stress task, including time-limited Raven’s matrices trials with performance feedback. STAI-S, State form of Spielberger’s State-Trait Anxiety Inventory; SSCS-S, State Self-compassion Scale-Short form; PANAS, Positive Affect and Negative Affect Scale; SC, Self-Compassion; HR, Heart Rate; HRV, Heart Rate Variability.

This study was a 2 (group: self-compassion induction vs. control) by 2 (time: pre vs. post) mixed factorial design. Participants completed the State form of Spielberger’s State-Trait Anxiety Inventory (24), the State Self-compassion Scale-Short form (25), the Perceived stress, and the Positive Affect and Negative Affect Scale (26) both before and after the induction. Participants performed a computerized stress-inducing task, during which ECG data were recorded (**Figure 1a**).

The stress-inducing task comprising 13 challenging items from the Raven’s Standard Progressive Matrices (Chinese City Edition). Specifically, items B12, C10, C12, D9-D12, E7–12 were included (27, 28). At the beginning of the task, participants were informed that the test could accurately assess their intelligence and may predict their future success. The task consisted of 13 trials, with each trial started with a 1-second fixation, followed by the presentation of a reasoning item with a 20-second countdown, during which participants were instructed to think and respond. After the countdown, feedback was presented for 6 seconds, randomly labelling 2 items as “Correct” and the remaining as “Wrong”. At the end of the task, the computer screen displayed the message, ‘Unfortunately, you failed!’ (**Figure 1b**).

### 2.3 Self-compassion and control induction

Participants completed a writing-based compassionate self-talk protocol adapted from validated procedures (19, 20). Participants were instructed to generate four compassionate phrases intended to comfort others facing negative life events. They were encouraged to write statements in their own words, fostering self-compassion in response to life stress. The underlying rationale for this approach is that by generating their “own” familiar compassionate statements for a close friend, individuals may internalize these phrases, which can subsequently promote self-compassion when they are faced with a life stressor. Instructions were as follows:

“*Now, please imagine that one of your closest friends is going through something difficult. He or she is worried, anxious, even miserable, and feels bad about him/herself. Please write four sentences to comfort and encourage him/her, and try to express your compassion, understanding and unconditional acceptance for your friend. Keep the sentences to fifteen words*.”

Participants in both conditions were allocated 15 minutes to engage with their respective tasks. In the self-compassion condition, participants generated four compassionate phrases to comfort a friend and were instructed to visualize the supportive scenario. In the control condition, participants were provided with four neutral sentence starters—’A bird is flying over the roof,’ ‘The store is selling fruits,’ ‘A TV is on air,’ and ‘The water level rose after a rain’—and were asked to complete these sentences using their imagination. Participants in both groups spent the duration of the 15-minute block writing their phrases, mentally imagining the scenes, and repeatedly reading their completed statements aloud until the time elapsed.

### 2.4 Measurements

*State Self-compassion Scale-Short Form (SSCS-S)*. This scale comprises 6 items, with 3 being reverse-scored. This self-reported measure is designed for assessing state self-compassion (25). Items are rating scores from 1 (almost) to 5 (almost always). The Cronbach’s α of the Chinese version is 0.68.

*State form of Spielberger’s State-Trait Anxiety Inventory (STAI-S).* This is a self-reported measure designed to assess measure state anxiety (24). This subscale consists of 20 items. All items are rated on a four-point scale ranging from 1 (not at all) to 4 (very much so). Total score ranges from 20 to 80, with higher scores indicating greater levels of anxiety. Chinese version is well-validated, with test-retest reliability of 0.88 (29).

*Positive and Negative Affect Schedule (PANAS).* The PANAS is used to assess emotion states and contains two subscales: positive affect subscale and negative affect subscale. Each subscale includes 10 emotion-related adjectives to assess positive or negative emotions (30). Participants rated according to how they felt over the last few days using a 5-point scale (1 = very slightly to 5 = most of the time). Higher scores indicate higher positive or negative affect. The Chinese version of the scale has demonstrated good reliability and validity, with a Cronbach’s α of 0.82 (25).

*Perceived stress.* Participants reported how much stress they are experiencing currently using a 10-point Likert scale from 1 (almost no) to 10 (almost worst) (31).

### 2.5 ECG acquisition

ECG signals were recorded using a BITalino (r)evolution Board Kit BT (BITalino, Portugal) (http://bitalino.com/en/). Three disposable Ag/AgCl electrodes were utilized: two were positioned bilaterally on the chest near the clavicles, and one was placed at the lower edge of the left rib cage. Signals were digitized at 1000 Hz sampling rate via OpenSignals (r)evolution software (v.2017, BITalino, Portugal) running on a separate computer.

### 2.6 Data analysis and statistics

#### 2.6.1 Heart rate analyses

Inter-beat interval (IBI) series for the ECG data were obtained using the Pan-Tompkins algorithm, which identifies the R wave peak as the fiducial point (32). Visual inspection and manual editing were performed to remove artifacts in accordance with established guidelines (33). The IBI series were subsequently converted into beats per minute (BPM) series. Continuous data were segmented based on the onset of feedback, ranging from -1 to 6 seconds. Trials associated with “Wrong” feedback were retained and subjected to baseline correction for each trial (from -1 to 0 seconds, with time 0 marking feedback onset). This approach is intended to account for individual variations in baseline heart rate and to capture the short-term dynamics of event-related heart rate changes (34). For each participant, heart rate data were averaged across trials, and the area under the curve (AUC) was calculated using the linear trapezoidal rule to quantify the event-related heart rate changes during negative feedback presentation.

#### 2.6.2 Heart rate variability analyses

Both HR and HRV analyses were performed on the same segmented (-1 to 6 s) data. After identifying and verifying the IBI, R-R intervals were derived and linearly interpolated to 4 Hz to obtain evenly sampled signals (35). The interpolated R-R interval waveform was subsequently detrended using a high-pass filter with a cutoff frequency of 0.02 Hz (36). A time-varying autoregressive (TVAR) model was applied for the time-frequency analysis of heart rate variability (HRV) (37, 38). This model offers the advantage of providing smooth spectral components and accurate power spectrum estimation and has previously been applied to investigate beat-to-beat spectra during acute stress (19). The model order was established at 12 based on existing literature (37). The normalized high frequency (nHF) HRV parameter was calculated over time for each subject, representing the high frequency component (0.15-0.4 Hz) as a relative value against the total power, excluding the very low frequency component (0-0.04 Hz) (39). nHF-HRV generally indicates the balance between sympathetic and parasympathetic nerve activities (39). Trials corresponding to the feedback of ‘Wrong’ (-1 to 6 seconds) were analyzed and baseline corrected for each trial (-1 to 0 seconds, where time 0 denotes the onset of the feedback).

Differences in heart rate changes between the SC condition and the control condition were then assessed. Given the dynamic nature of heart rate, we aimed to identify specific time windows where these differences were statistically significant. To accomplish this, we utilized a sliding window approach with a window size of 500 ms and a step size of 50 ms (40–42). Within each window, heart rate change was calculated as the difference in the area under the curve (AUC) between the SC condition and control condition (40). Because this procedure involved a large number of overlapping tests across time, p values across windows were corrected for multiple comparisons using false discovery rate (FDR) correction.

#### 2.6.3 Statistical analyses

Using SPSS (version 23; IBM Corp, Armonk, NY), we first compared baseline demographic and clinical characteristics between groups employing independent sample t-tests for continuous variables and χ² tests for categorical variables. We then examined group differences between the SC condition and control condition in changes (post - pre) in state anxiety, state self-compassion, positive affect, negative affect, and perceived stress following the stress-inducing task using independent sample t-tests. Additionally, independent t-tests were conducted on the changes in heart rate and nHF-HRV between the two groups to assess physiological changes. Pearson correlation analyses were performed to explore the relationship between heart rate changes and behavioral changes, thereby investigating the body-mind connection.

## 3 Results

### 3.1 Demographic and descriptive analysis

The demographics and baseline measures of participants are presented in **Table 1**. Independent samples t-tests confirmed no significant between-group differences in baseline anxiety (*t* (44) = 1.60, p = 0.12) or depression scores (*t* (44) = -0.63, p = 0.54). 

**Table 1:** Demographics and baseline psychological measures of the SC group and control group.

Characteristic	Total (n = 46)	SC group (n = 23)	Control group (n = 23)	p value^a^
Age, years: mean (SD)	33.93(9.38)	38.52(7.61)	29.34(8.86)	0.00
Gender, n (%) Female Male	34(73.9) 12(26.1)	17(73.9) 6(26.1)	17(73.9) 6(26.1)	1.0
Marital Status, n (%) Married Single/Separated	26(56.5) 20(43.5)	17(73.9) 6(26.1)	9(39.1) 14(60.9)	0.02
Education, n (%) Secondary High school University degree Postgraduate degree	5(10.9) 10(21.7) 27(58.7) 4(8.7)	4(17.4) 6(26.1) 11(47.8) 2(8.7)	1(4.3) 4(17.4) 16(69.6) 2(8.7)	0.37
Employment, n (%) Unemployed/Housewife/Retired Employed	3(6.5) 43(93.5)	1(4.3) 22(95.7)	2(8.7) 21(91.3)	1.0
Duration of patients had general anxiety, years, n (%) 0.5-1 1-3 3-5 5-10 ≥10	22(47.8) 19(41.4) 3(6.4) 1(2.2) 1(2.2)	9(39.1) 10(43.4) 2(8.7) 1(4.3) 1(4.3)	13(56.5) 9(39.1) 1(4.3) 0 (0) 0(0)	0.43
HAMA score, mean (SD)	22.3(5.82)	23.65(7.07)	20.96(3.94)	0.12
PHQ-9 score, mean (SD)	10.74(5.15)	10.26(5.15)	11.22(5.21)	0.54

SC group, self-compassion group; HAMA, Hamilton Anxiety Rating Scale; PHQ-9, Patient Health Questionnaire-9.

a : Estimated by χ2 test for categorical variables, and independent t-tests for continuous variables.

### 3.2 Behavioral outcomes

**Figure 2:**
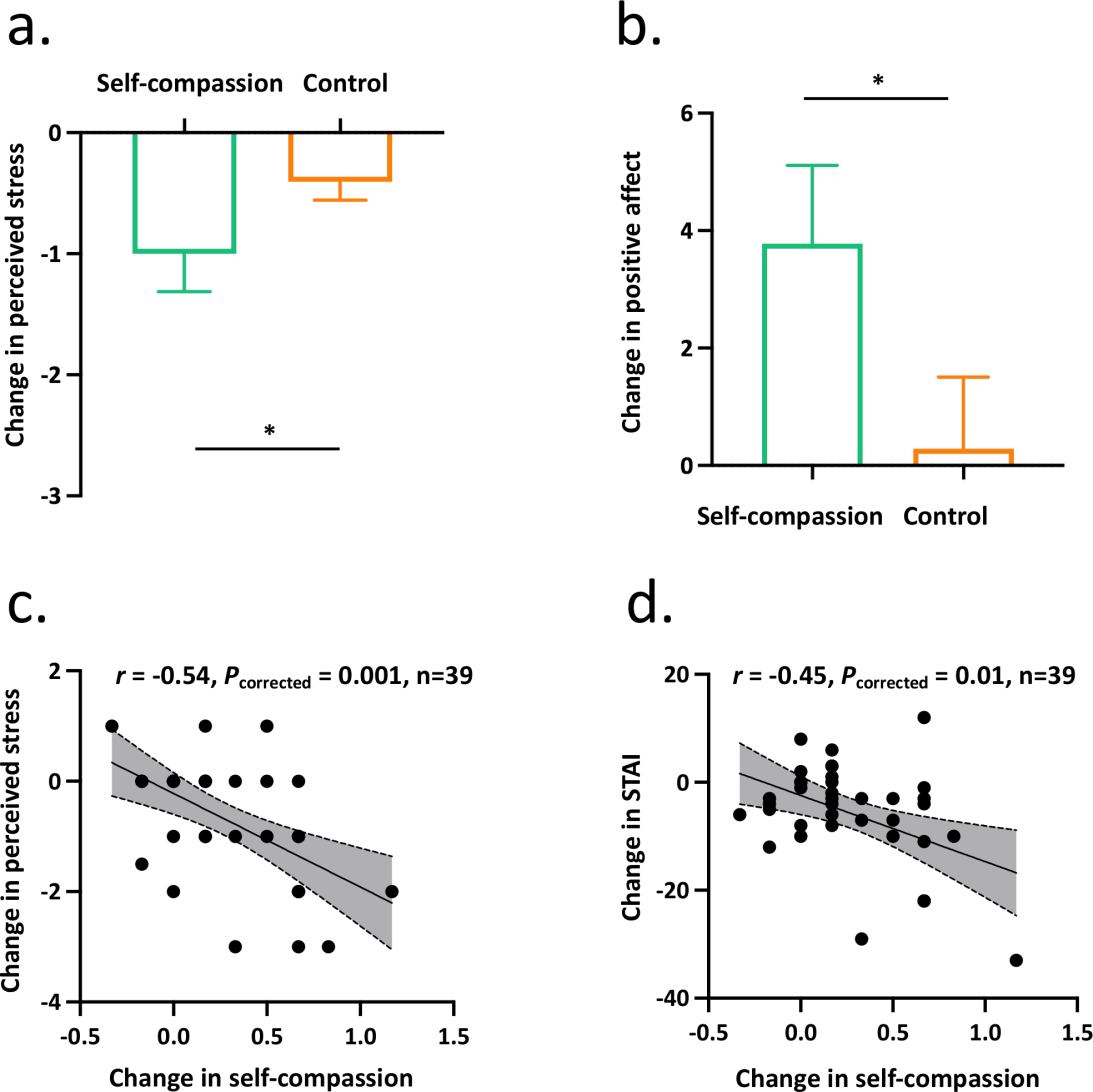
Intervention effects on behavioral outcomes. **(a)** Results indicated greater perceived stress reduction in the self-compassion group (*t_(44)_* = -2.09, *p* = 0.043, *d* = 0.62). **(b)** The self-compassion group showed increased positive affect (*t_(44)_* = 2.29, *p* = 0.028, *d* = 0.67). **(c, d)** Significant correlations between state self-compassion changes and reduced perceived stress (*r* = -0.54, *p* = 0.001) and anxiety (*r* = -0.45, *p* = 0.01). Dots represent individual participants; shaded areas indicate 95% confidence intervals.

The analysis of changes in perceived stress revealed a significant difference between the self-compassion group and the control group (*t _(44)_* = -2.09, p = 0.043, d = 0.62). Notably, the self-compassion group demonstrated a significantly greater reduction in perceived stress (*ΔM* = -0.91 ± 1.20) compared to controls (*ΔM* = -0.24 ± 0.98) (**Figure 2a**).

A significant group difference emerged in positive affect changes (self-compassion: *ΔM* = 4.70 ± 7.14vs. control: *ΔM* = 0.43 ± 5.38; *t _(44)_* = 2.29, *p* = 0.028, *d* = 0.67), with larger improvements in the intervention group (**Figure 2b**). Meanwhile, no significant between-group difference was observed in the changes of negative affect (*t _(39)_* = 1.41, *p* = 0.17).

Neither state anxiety (*t _(44)_* = -0.97, *p* = 0.337) nor state self-compassion (*t _(44)_* = 0.9, *p* = 0.375) showed significant intervention effects. Correlational data indicated that increased state self-compassion was strongly associated with reduced perceived stress (*r* = -0.54, *df* = 38, *p* = 0.001) (**Figure 2c**), as well as with lower state anxiety (*r* = -0.45, *df* = 38, *p* = 0.01) (**Figure 2d**). The measures for each group assessed at pre and post were presented in **Table 2**.

**Table 2:** Means and standard deviations for outcome measures.

		SC group (n = 23)	Control group (n = 23)	p value^a^
Measure	Time	M(SD)	M(SD)	
STAI-S	pre	16.35(4.59)	15.74(4.58)	0.66
	post	13.82(4.27)	14.09(3.73)	0.83
SSCS-S	pre	2.72(0.70)	2.72(0.52)	0.97
	post	3.05(0.87)	2.93(0.51)	0.59
PA	pre	24.35(6.11)	23.22(4.81)	0.49
	post	29.04(6.91)	23.65(5.20)	0.005
NA	pre	28.71(8.81)	28.52(9.66)	0.95
	post	21.50(7.74)	18.95(7.53)	0.29
Perceived stress	pre	5.96(2.20)	5.61(2.10)	0.59
	post	5.04(2.18)	5.37(1.85)	0.59

SC, Self-compassion Group; SSCS-S, State Self-compassion Scale-Short Form; STAI-S, State form of Spielberger’s State-Trait Anxiety Inventory; PA, Positive Affect; NA, Negative Affect.

a : Estimated by independent t-tests.

### 3.3 Heart rate and heart rate variability

**Figure 3:**
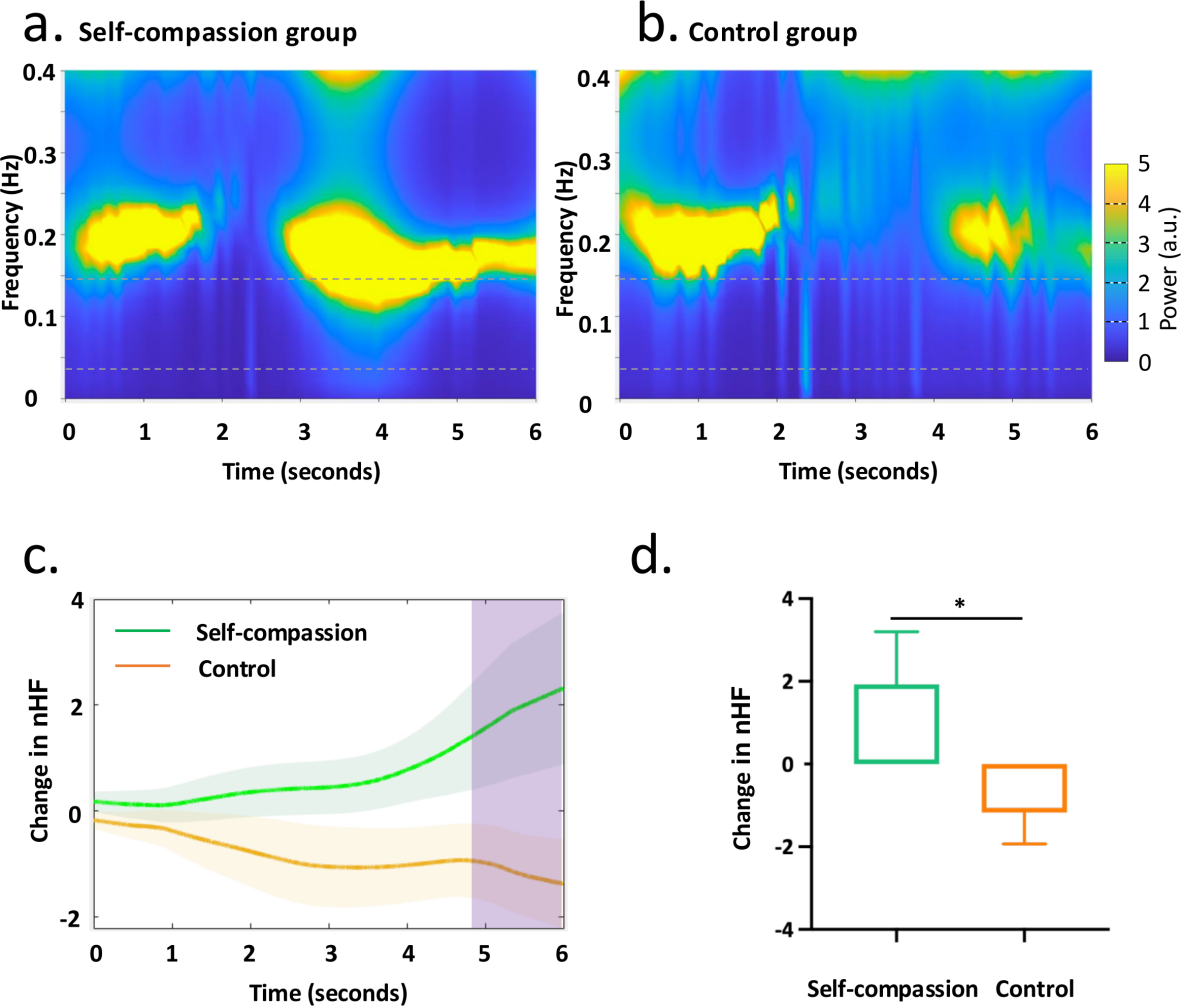
Time-frequency analysis of HRV during negative feedback. **(a, b)** Representative time-frequency maps for self-compassion **(a)** and control **(b)** groups, with dashed lines marking the boundaries of very low (0-0.04 Hz), low (0.04-0.15 Hz), and high (0.15-0.4) frequency bands. **(c)** Significant group difference in nHF-HRV during 4.8-6s post-feedback (highlighted in dark). **(d)** The self-compassion group exhibited a higher nHF-HRV change compared to the control condition (*t _(37)_* = 2.15, *p _corrected_* = 0.038), with asterisk denoting *p*_FDR_ < 0.05. nHF denotes normalized HF-HRV.

No significant between-group differences emerged in heart rate changes during negative feedback periods across the task. There is a group difference in nHF-HRV during the 4.8 to 6 seconds post-feedback window (*t _(37)_* = 2.15, *p _corrected_* = 0.038), indicating enhanced parasympathetic engagement in the self-compassion group see **Figures 3a, b** for representative maps (**Figures 3c, d**).

Pearson correlation analyses were conducted to explore the relationship between nHF-HRV changes and all behavioral outcomes, including changes in state anxiety, state self-compassion, positive affect, negative affect, and perceived stress. No significant relationships were observed (*p*_s_ > 0.05). All the correlation data were presented in the **Supplementary Table 1**.

## 4 Discussion

This randomized controlled trial provides preliminary evidence that a single-session self-compassion intervention may enhance stress resilience in GAD through associated emotional and autonomic improvements. Specifically, the intervention group exhibited reduced perceived stress, increased positive affect, and elevated HF-HRV during cognitive stress exposure – suggesting potentially enhanced parasympathetic regulation. By contrast, no significant group differences were observed for state anxiety, negative affect, or state self-compassion. Accordingly, the present findings support a short-term effect on some affective outcomes rather than a broad immediate therapeutic effect.

An important finding is the increased HRV in the self-compassion group compared to controls. Our observation of HF-HRV augmentation within a specific, limited temporal interval (4.8-6s post-feedback) aligns with neurovisceral integration models (43), where self-compassion may strengthen prefrontal inhibition over amygdala-driven autonomic arousal. From a physiological and medical perspective, this brief manifestation suggests that the intervention facilitates acute autonomic flexibility, a rapid, adaptive parasympathetic response, during stress rather than inducing stable, long-term enhancements in cardiovascular health. Meanwhile, given that HRV is widely recognized as an objective index of emotional regulation (44), our results suggest that self-compassion training may transiently enhance cardiac vagal activity—a marker of improved momentary autonomic regulation and emotional control (10, 45). The dynamic analysis of HRV indicated that the control group maintained relatively stable HRV, while the self-compassion group demonstrated a continuous increase over time, with significant differences emerging during the later stages of the negative feedback. The delayed HRV increase suggests a sustained regulatory process – potentially reflecting gradual engagement of newly acquired self-compassion strategies during prolonged stress appraisal. Notably, while state anxiety remained unchanged, the strong correlation between self-compassion gains and stress reduction (*r* = -0.54) supports theoretical accounts positing self-compassion as a transdiagnostic resilience factor (46).

The observed reduction in perceived stress and enhancement of positive affect among self-compassion participants align with previous findings demonstrating the benefits of self-compassion interventions in individuals with GAD (12, 41, 42). Importantly, our data extend the current literature by providing evidence that even a brief self-compassion exercise can be feasibly implemented to alleviate GAD symptoms. The lack of a significant increase in state self-compassion following the exercise is noteworthy; however, the correlational analysis supports the notion that higher self-compassion is associated with lower stress and anxiety, consistent with behavioral models positing that self-compassion facilitates adaptive coping in various mental disorders (5, 47, 48).

Contrary to some previous studies (18, 49, 50), we did not observe significant changes in state self-compassion, state anxiety, or negative affect. This discrepancy may be attributed to the inherent challenges GAD patients face in initiating self-care practices, potentially due to elevated levels of shame, self-criticism, and anxiety (51–53). Although the self-compassion exercise provided temporary warmth and comfort through a self-dialogue format, the chronic nature of GAD may render a single session insufficient to produce significant changes in entrenched symptoms. Moreover, the compassionate self-talk exercise involved generating phrases to comfort a friend rather than directly addressing the self. While this is a validated method intended to prime a broader compassionate mindset that participants can internalize, it is possible that the manipulation might activate empathic, prosocial affect, motional distancing or perspective-taking instead of specific self-directed compassion. This distinction might partly explain the lack of a significant main effect on the state self-compassion scale.

This study has several limitations. First, although an *a priori* power analysis was conducted, the final sample size for the HRV analyses was relatively small following necessary data exclusions for physiological artifacts. This limits the statistical power of our findings and increases the potential for overestimating the significant effects. Consequently, the observed transient autonomic changes should be interpreted with caution. Second, baseline differences in age and marital status may have influenced both behavioral and physiological outcomes, and these group imbalances should be considered when interpreting the results. Future research should employ stratified randomization or mixed-effects models to better isolate the specific effects of the intervention from demographic confounders. Third, for ethical reasons, medication use and concurrent psychotherapy were not controlled, although randomization and baseline comparisons suggested minimal group differences in these variables. Fourth, the reliance on literacy for the self-compassion writing exercise may limit the generalizability of this intervention. Future studies should employ multi-session designs to test dose-response relationships and integrate neuroimaging (e.g., fMRI during self-compassion recall) with HRV (54, 55). Fifth, it is important to note that our HRV parameters were computed over very short time segments (-1 to 6 seconds) using a TVAR model. While the TVAR model is capable of providing continuous beat-to-beat spectra during acute stress, traditional HRV measures typically require longer recordings (often 1 to 5 minutes) to ensure stable spectral estimates. Consequently, the physiological interpretation of the observed HF-HRV differences as strictly reflecting parasympathetic modulation should be made cautiously, as short-term estimates may contain inherent instability. Finally, regarding potential differences in cognitive demands between the two conditions, we designed the control task to closely mirror the experimental task. By asking the control group to creatively complete neutral sentences and imagine the corresponding scenes, we aimed to balance the cognitive load, creativity, and time engagement across both groups. Nevertheless, we acknowledge that generating emotionally salient, compassionate content may inherently require a different type or degree of cognitive resource compared to neutral content generation. Future studies could incorporate subjective or objective measures of cognitive effort to further rule out any subtle confounding effects of task complexity.

This study has several implications. First, a brief self-compassion exercise appears to improve both emotional and physiological responses to stress in GAD patients, offering a valuable adjunctive treatment option. Second, the simplicity and accessibility of self-compassion induction make it suitable for both inpatient and outpatient settings and directly adaptable to digital platforms, potentially facilitating its integration into daily routines. Finally, brief self-compassion exercises may serve as an introductory step toward long-term self-compassion programs, thereby motivating patients to pursue more extensive psychotherapeutic interventions tailored to their needs. In line with this, future studies might consider integrating HRV biofeedback with self-compassion training to further monitor and optimize autonomic intervention efficacy.

In conclusion, brief self-compassion training shows potential to confer acute emotional and autonomic benefits in GAD. By prioritizing physiological resilience alongside subjective stress relief, it could offer a pragmatic tool to augment existing therapies by targeting maladaptive autonomic patterns.

## Data Availability

The raw data supporting the conclusions of this article will be made available by the authors, without undue reservation.
