# Supplementary material for: Compassionate self-talk enhances autonomic flexibility during cognitive stress in generalized anxiety disorder: a randomized controlled trial with HRV evidence
Source: Front Psychiatry. 2026 Jun 16;17:1795990. doi: 10.3389/fpsyt.2026.1795990 (PMC13317479; doi:10.3389/fpsyt.2026.1795990)
Supplement: Supplementary file 1 [file Table1.docx]

Supplementary Material

# Supplementary Tables

# Supplementary Table 1. Correlation coefficients of variables in all patients

| Variables | 1 | 2 | 3 | 4 | 5 | 6 | 7 | 8 |
| --- | --- | --- | --- | --- | --- | --- | --- | --- |
| 1. changes in HRV | - |  |  |  |  |  |  |  |
| 2. HAMA | 0.28 | - |  |  |  |  |  |  |
| 3. PHQ-9 | -0.13 | 0.33* | - |  |  |  |  |  |
| 4. changes in STAI-S | -0.04 | 0.03 | -0.01 | - |  |  |  |  |
| 5. changes in SCS | -0.12 | -0.06 | -0.01 | -0.45** | - |  |  |  |
| 6. changes in PS | 0.06 | -0.13 | -0.18 | 0.45** | -0.54** | - |  |  |
| 7. changes in Positive affect | -0.00 | 0.02 | -0.21 | 0.17 | 0.01 | -0.14 | - |  |
| 8. changes in Negative affect | 0.04 | -0.11 | -0.08 | 0.20 | -0.16 | 0.24 | -0.31 | - |

*Note.* HRV, heart rate variability; HAMA, Hamilton Anxiety Rating Scale; PHQ-9, Patient Health Questionnaire-9; STAI-S, State form of Spielberger’s State-Trait Anxiety Inventory; PS, Perceived Stress.

n=39, **P* < 0.05, ***P* < 0.01.
